# Possible Effect of Climate Change on Surface-Water Photochemistry: A Model Assessment of the Impact of Browning on the Photodegradation of Pollutants in Lakes during Summer Stratification. Epilimnion vs. Whole-Lake Phototransformation

**DOI:** 10.3390/molecules25122795

**Published:** 2020-06-17

**Authors:** Federico Calderaro, Davide Vione

**Affiliations:** Department of Chemistry, University of Torino, Via Pietro Giuria 5, 10125 Torino, Italy; federico.calderar@edu.unito.it

**Keywords:** surface–water photochemistry, environmental modeling, pollutant attenuation, lake dynamics, emerging pollutants, contaminants of emerging concern, effects of climate change

## Abstract

Water browning in lakes (progressive increase of the content of chromophoric dissolved organic matter, CDOM) has the potential to deeply alter the photodegradation kinetics of pollutants during summer stratification. Browning, which takes place as a consequence of climate change in several Nordic environments, causes the thermocline to be shallower, because higher CDOM decreases the penetration of sunlight inside the water column. Using a model approach, it is shown in this paper that pollutants occurring in the epilimnion would be affected differently depending on their main photodegradation pathway(s): almost no change for the direct photolysis, slight decrease in the degradation kinetics by the hydroxyl radicals (^•^OH, but the resulting degradation would be too slow for the process to be effective during summer stratification), considerable decrease for the carbonate radicals (CO_3_^•−^), increase for the excited triplet states of CDOM (^3^CDOM*) and singlet oxygen (^1^O_2_). Because it is difficult to find compounds that are highly reactive with CO_3_^•−^ and poorly reactive with ^3^CDOM*, the degradation rate constant of many phenols and anilines would show a minimum with increasing dissolved organic carbon (DOC), because of the combination of decreasing CO_3_^•−^ and increasing ^3^CDOM* photodegradation. In contrast, overall photodegradation would always be inhibited by browning when the whole water column (epilimnion + hypolimnion) is considered, either because of slower degradation kinetics in the whole water volume, or even at unchanged overall kinetics, because of unbalanced distribution of photoreactivity within the water column.

## 1. Introduction

The photoinduced transformation processes are important pathways for the attenuation of biorecalcitrant contaminants that occur in surface waters [1,2]. These processes are usually divided into direct photolysis, where the pollutant absorbs sunlight and is transformed as a consequence, and indirect photochemistry [3,4]. In the latter case, sunlight is absorbed by naturally occurring compounds (photosensitizers) such as the chromophoric dissolved organic matter (CDOM), nitrate, and nitrite [5]. The absorption of radiation by photosensitizers causes the generation of reactive transient species such as the hydroxyl radical (^•^OH), singlet oxygen (^1^O_2_), and CDOM triplet states (^3^CDOM*) [6,7]. Moreover, the oxidation of inorganic carbon species (HCO_3_^−^ by ^•^OH; CO_3_^2−^ by ^•^OH and ^3^CDOM*) yields the carbonate radical, CO_3_^•−^ [8]. All the mentioned transients (^•^OH, ^1^O_2_, ^3^CDOM*, and CO_3_^•−^) react at a variable extent with pollutants to induce their degradation [5,9].

Photochemical reactions in surface waters are affected by several environmental factors and environmental modifications, among which climate change plays a major role [10,11]. Among the climate-induced changes to water chemistry, variations in the contents of both dissolved organic matter (DOM) and its chromophoric fraction (CDOM) have the highest potential to affect photoreactions [12,13]. Increasing average precipitation in several regions of the world, or an increased likelihood of extreme events even at constant average precipitation, is enhancing transport by runoff of organic material from soil to surface waters [14,15,16,17]. The result is an increase in surface-water (C)DOM levels (browning), which could affect photoreactions by enhancing processes triggered by ^3^CDOM* and ^1^O_2_, over those induced by ^•^OH, CO_3_^•−^ and the direct photolysis [11,13]. The increase of (C)DOM is not a general occurrence, and in some cases the opposite trend has been reported [18]. However, compared to browning, the underlying mechanisms of (C)DOM decrease have not yet been clarified.

In addition to water chemistry, (C)DOM variations affect water dynamics in certain circumstances such as, most notably, summer stratification in lakes [19]. Lakes that are deep enough can become stratified during summer, because sunlight heats the lake surface and makes the surface water warmer and less dense. Therefore, a warmer and less dense surface layer (epilimnion) floats over the colder and denser bottom water (hypolimnion). Mass exchange between the two compartments takes place only by diffusion, thus epilimnion and hypolimnion evolve separately. Epilimnion and hypolimnion are separated by the thermocline, which is defined as the (very thin) water layer where the steepest temperature gradient with depth is observed [20].

CDOM is the key sunlight absorber in lake water below 500 nm, and sunlight penetration in the water column is higher if the CDOM levels are lower [21]. In the presence of high CDOM, limited sunlight penetration reduces the portion of the water column that can be heated by sunlight, and shallower thermocline is produced as a consequence. The depth of the epilimnion coincides with the depth of the thermocline, thus one expects an inverse relationship between CDOM levels and epilimnion depths [19].

From the point of view of pollutant photodegradation, increasing CDOM levels are often detrimental to decontamination, while decreasing depth is favorable [22]. Therefore, the relationship between CDOM and epilimnion depth (low CDOM ⇔ deep epilimnion; high CDOM ⇔ shallow epilimnion) may have non-trivial impacts over the ability of epilimnion water to induce photochemical attenuation of pollutants. In this work, the photodegradation kinetics of two photochemically labile pharmaceuticals and a more photostable artificial sweetener are modeled under summertime sunlight conditions, in reasonable epilimnion waters as far as CDOM levels and thermocline depths are concerned. One of the pollutants undergoes degradation mainly by direct photolysis (diclofenac, DICL) [23], another mainly by indirect photochemistry (paracetamol, a.k.a. acetaminophen; or *N*-acetyl-para-aminophenol, APAP) [24]. The third pollutant (acesulfame k, ACEK) is degraded only by a peculiar indirect photochemistry pathway (^•^OH reaction) [25]. Photodegradation in the epilimnion is relevant to the pollutants that reach the lake surface by, e.g., soil runoff or shallow input streams [26]. Implications are considered, as well, for the photodegradation of pollutants that are homogeneously distributed within the whole water column before the onset of stratification.

## 2. Theoretical Background

The photochemistry modeling software used in this work predicts the pseudo-first order degradation rate constants of pollutants and the associated half-life times, as a function of sunlight irradiance, water chemistry and depth, as well as of the pollutant photoreactivity parameters (absorption spectrum, direct photolysis quantum yield, and second-order reaction rate constants with ^•^OH, CO_3_^•−^, ^1^O_2_, and ^3^CDOM*). The following scheme of photochemical reactions is used in the model, upon which the software is based [9,27]

NO_3_^−^ + hν + H^+^ → ^•^NO_2_ + ^•^OH (1)

NO_2_^−^ + hν + H^+^ → ^•^NO + ^•^OH (2)

CDOM + hν →→→ ^•^OH (3)

(4) $$\mathrm{CDOM}+h\nu \;\overset{ISC}{\to }{\;}^{3}\mathrm{CDOM}*\;\overset{O_{2}}{\to }\;\mathrm{CDOM}+{\;}^{1}O_{2}$$

^•^OH + HCO_3_^−^ → H_2_O + CO_3_^•−^ (5)

^•^OH + CO_3_^2−^ → OH^−^ + CO_3_^•−^ (6)

^3^CDOM* + CO_3_^2−^ → CDOM^•−^ + CO_3_^•−^ (7)

^•^OH + DOM → Products (8)

CO_3_^•−^ + DOM → Products (9)

^3^CDOM* + DOM → Products (10)

(11) $${}^{1}O_{2}\;\overset{H_{2}O}{\to }\;O_{2}$$

Note that ISC = inter-system crossing, and that the reaction mechanisms yielding ^•^OH from irradiated CDOM (Reaction (3)) are not yet completely elucidated [28,29]. Still, it is possible to reliably quantify ^•^OH photogeneration from CDOM [30]. Reactions (1)–(3) represent formation of reactive species, while Reactions (8)–(11) describe scavenging/quenching processes. Reaction (4) depicts ^3^CDOM* photogeneration and scavenging, as well as ^1^O_2_ formation. Reactions (5)–(7) are scavenging processes for ^•^OH/^3^CDOM* that lead to CO_3_^•−^ generation. All these reactions explain why the photochemistry of surface waters is affected by water chemistry. Another important parameter is water depth because, all other things being equal, shallower waters are more thoroughly illuminated by sunlight [31].

In the case of stratified lakes, it is possible to consider the photochemistry of the epilimnion, by taking the depth of the thermocline as the water depth required by the photochemical model [11]. Heating by sunlight is essential for the lake water to become stratified during summer, and the penetration depth of sunlight plays a key role in defining the depth of the thermocline [20]. The ability of the lake water to absorb sunlight can be determined as the absorbed photon flux, *P*_a_. According to a Lambert-Beer approach, the *P*_a_ of the lake water (units of Einstein L^−1^ s^−1^) can be expressed as [32]

(12) $$P_{a}=\int_{\lambda }p{}^{\circ}(\lambda )\;[1-10^{-100\;A_{1}(\lambda )\;DOC\;d_{th}}]\;d\lambda$$

where *p*°(λ) (Einstein L^−1^ s^−1^ nm^−1^) is the incident (over-water) spectral photon flux density of sunlight at the wavelength λ [nm], *A*_1_(λ) (L mg_C_^−1^ cm^−1^) is the specific absorbance of the lake water (i.e., the absorbance value per unit DOC and 1-cm optical path length), DOC (mg_C_ L^−1^) is the dissolved organic carbon, *d*_th_ (m) is the depth of the thermocline, and 100 is the conversion factor between meters and centimeters [9].

The DOC includes both sunlight-absorbing and non-absorbing organic compounds, and an increase in the DOC usually increases the ability of the lake water to absorb sunlight [13,14,30]. Therefore, the higher is the DOC, the lower is expected to be *d*_th_ (indeed, Equation (12) contains the product DOC × *d*_th_, and *P*_a_ does not change if DOC × *d*_th_ is constant) [19]. Coherently, in a study that considered a number of lowland lakes, it has been reported that the product DOC × *d*_th_ ranged between 30–60 m mg_C_ L^−1^ during summer stratification [33]. In most surface waters, the value of the specific absorbance has as an exponentially decaying trend with increasing wavelength [34], and a suitable approximation for λ = 290–500 nm has been reported as follows: $A_{1}(\lambda )=0.45\;e^{-0.015\;\lambda }$ [30]. By including the latter relationship in Equation (12), and with the product DOC × *d*_th_ in the range of 30–60 m mg_C_ L^−1^, one finds that the epilimnion lake water would be able to absorb 96–99.9% of the incident sunlight photons in the wavelength interval of 290–500 nm. Such an extensive ability to absorb sunlight looks reasonable for a lake epilimnion.

**Figure 1:**
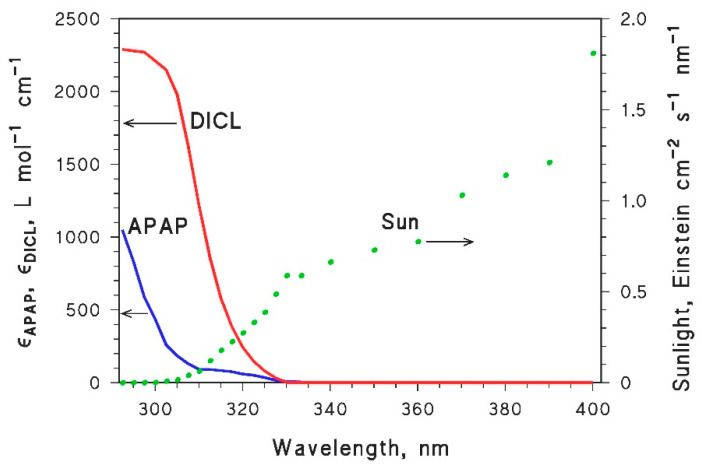
Left *y*-axis: Absorption spectra (molar absorption coefficients) of paracetamol (APAP) and diclofenac (DICL) [23,24]. Right *y*-axis: Spectral photon flux density of sunlight, incident on the water surface. In mid-latitude conditions, this value of spectral photon flux density can be observed in July in mid-morning or mid-afternoon [27,39]. Note that ACEK does not absorb radiation at λ > 280 nm [25], thus it does not undergo direct photolysis under sunlight irradiation.

In this work, three model pollutants (APAP, DICL and ACEK) were chosen because their photoreactivity parameters are known [23,24,25]. The direct photolysis quantum yields and the second-order reaction rate constants with ^•^OH, CO_3_^•−^, ^1^O_2_, and ^3^CDOM* are reported in Table 1. Moreover, Figure 1 compares the relevant absorption spectra with the spectrum of sunlight used in the model.

In previous works, it has been shown that this model approach is able to predict well the phototransformation kinetics of both APAP and DICL in surface freshwaters [23,24,35,36], as well as the low photolability of ACEK [25,37,38]. A comparison between model predictions and field data is provided in Table 2.

To see how the thermocline depths could vary when varying the DOC levels, three different values of the product DOC × *d*_th_ were tested (30, 45, and 60 m mg_C_ L^−1^), which are included in the range observed in the field [33]. The pseudo-first order degradation rate constants of DICL, APAP, and ACEK, the corresponding half-life times, and the importance of the different photodegradation pathways (direct photolysis and reaction with ^•^OH, CO_3_^•−^, ^1^O_2_, and ^3^CDOM*) were studied in the DOC interval of 2–30 mg_C_ L^−1^. Other water conditions were kept constant (10^−4^ mol L^−1^ NO_3_^−^, 10^−6^ mol L^−1^ NO_2_^−^, 10^−3^ mol L^−1^ HCO_3_^−^, 10^−5^ mol L^−1^ CO_3_^2−^).

## 3. Results and Discussion

### 3.1. Photodegradation in the Epilimnion

**Figure 2:**
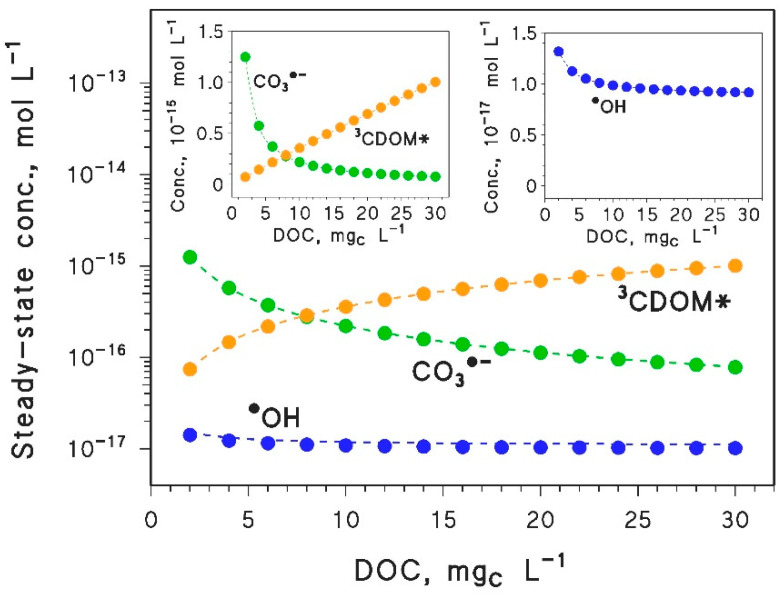
Modeled steady-state concentrations of ^•^OH, CO_3_^•^^−^, and ^3^CDOM* as a function of the DOC, for constant values of the product DOC × *d*_th_ = 45 m mg_C_ L^−1^. In the main figure panel, the *y*-axis has an exponential scale that gives insights into the orders-of-magnitude differences in concentrations. The same data are plotted with a linear *y*-axis scale in the two inserts, where one can appreciate the almost linear trend of [^3^CDOM*] with the DOC. Note that ^1^O_2_ has similar concentration values and similar trends as ^3^CDOM* [4]. Other water conditions: 10^−4^ mol L^−1^ NO_3_^−^, 10^−6^ mol L^−1^ NO_2_^−^, 10^−3^ mol L^−1^ HCO_3_^−^, and 10^−5^ mol L^−1^ CO_3_^2−^, sunlight irradiance as per Figure 1.

The contemporary DOC increase and *d*_th_ decrease has the potential to deeply alter photochemical processes. The DOC trends of the epilimnion steady-state concentrations of ^•^OH, CO_3_^•−^, and ^3^CDOM* are shown in Figure 2 for DOC × *d*_th_ = 45 m mg_C_ L^−1^ (the case of ^1^O_2_ is very similar to ^3^CDOM*). The low values of the steady-state concentrations are due to fast decay kinetics of these transient species, which are accounted for in the photochemical model (see Reactions (4)–(10)) [5].

It is shown in Figure 2 that, first of all, one has [^•^OH] « [CO_3_^•−^], [^3^CDOM*], which may be offset at variable extents by the fact that ^•^OH is a much stronger oxidant compared to CO_3_^•−^ or ^3^CDOM* [4,5,6]. Then, the steady-state [CO_3_^•−^] would strongly decrease with increasing DOC (one often observes [CO_3_^•−^] ∝ DOC^−2^ [11]). Actually, the dissolved organic matter (DOM, which increases with increasing DOC) directly scavenges CO_3_^•−^ (Reaction (9)). Moreover, DOM decreases the CO_3_^•−^ formation rate by scavenging ^•^OH and (to a lesser extent) ^3^CDOM* (Reactions (8) and (10)) [5,40]. The decrease of [^•^OH] with DOC is less marked, because ^•^OH is largely scavenged by DOM but is also partially generated by CDOM irradiation [5]. Finally, the increase of [^3^CDOM*] with increasing DOC ([^3^CDOM*] ∝ DOC) is due to the fact that the CDOM levels increase with increasing DOC, and CDOM irradiation is the only source of ^3^CDOM* [5,30].

**Figure 3:**
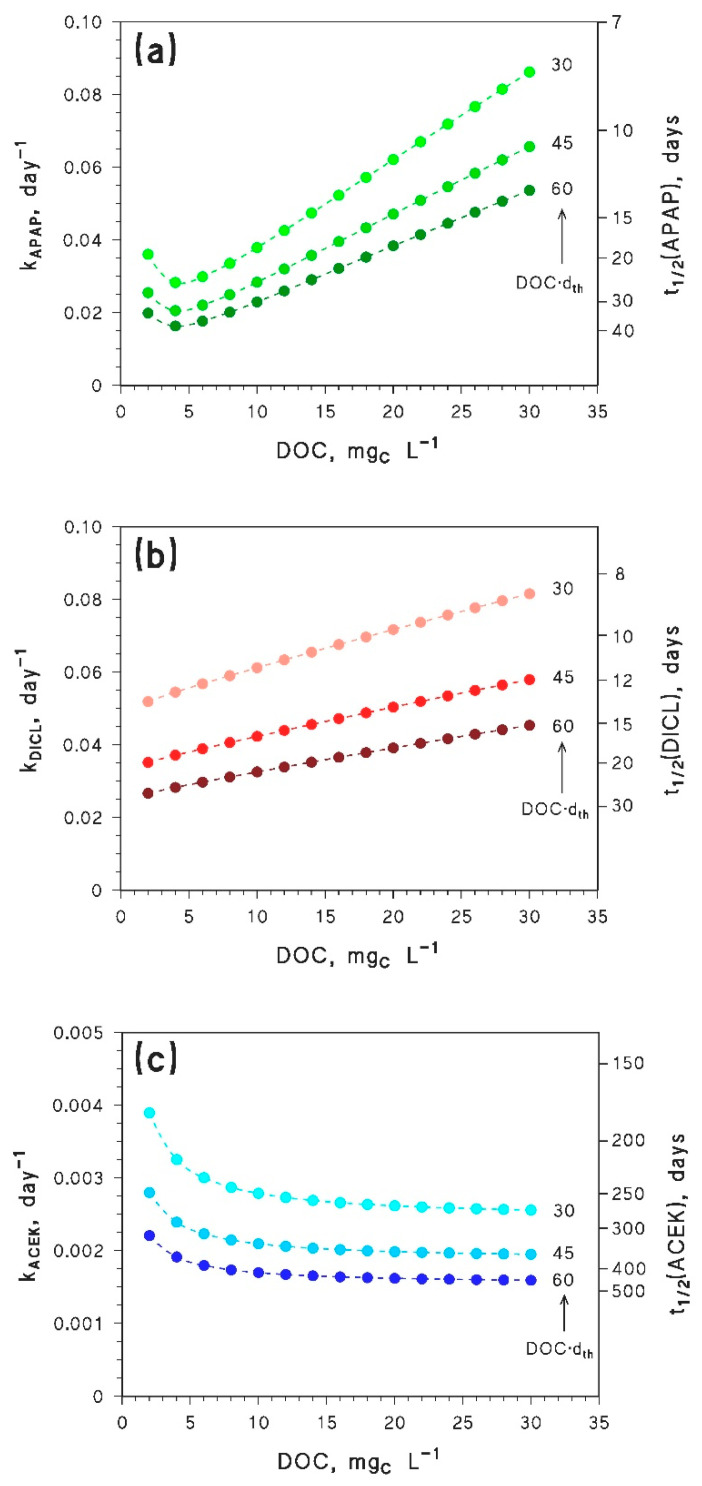
Modeled first-order rate constants and half-life times of (**a**) APAP, (**b**) DICL, and (**c**) ACEK in lake-water epilimnion, as a function of the DOC. The measure unit of the product DOC × *d*_th_ is (m mg_C_ L^−1^). Other water conditions: 10^−4^ mol L^−1^ NO_3_^−^, 10^−6^ mol L^−1^ NO_2_^−^, 10^−3^ mol L^−1^ HCO_3_^−^, 10^−5^ mol L^−1^ CO_3_^2−^. Photodegradation kinetics assumes mid-July irradiation conditions [27].

The results concerning the modeled photodegradation kinetics of the three compounds under study are reported in Figure 3 (3a: APAP; 3b: DICL, 3c: ACEK), as a function of the DOC and for values of the DOC × *d*_th_ product in the range between 30 and 60 m mg_c_ L^−1^. For all compounds, the predicted degradation at equal DOC is faster when the product DOC × *d*_th_ is lower. Indeed, lower DOC × *d*_th_ means shallower water for a given DOC value, and shallow waters have more chances than deep waters to be efficiently illuminated by sunlight [21,31].

In the case of APAP, the first-order rate constants show a minimum as a function of the DOC. In contrast, the DICL rate constants monotonically increase and the ACEK ones decrease with increasing DOC. Because APAP, DICL, and ACEK have different reactivity, the explanation of these trends has implications for the photochemical behavior of a much wider range of pollutants.

The predicted lifetimes of APAP range between 8–40 days, and would result in important APAP photodegradation in the epilimnion during the summer season. The minimum of the first-order rate constant (*k*_APAP_ vs. DOC) corresponds to conditions where photodegradation would be slower, which is predicted to take place at DOC ~ 4 mg_C_ L^−1^. Overall, the most favorable conditions to APAP photodegradation would be observed at high DOC. In the framework of the browning phenomenon (long-term, gradual DOC increase [14]), if the starting DOC was <4–5 mg_C_ L^−1^ it is very likely that APAP photodegradation kinetics would not change much with increasing DOC. Conversely, if the starting DOC was >4–5 mg_C_ L^−1^, browning would considerably enhance photodegradation in the epilimnion.

**Figure 4:**
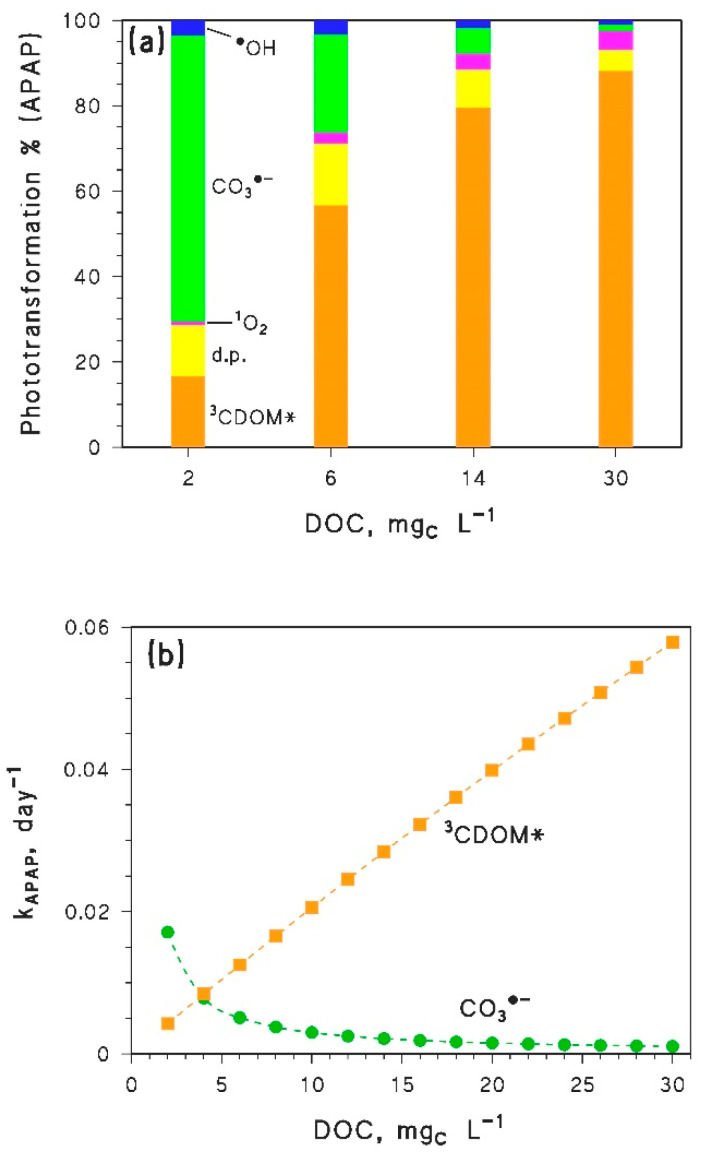
(**a**) Modeled contributions of ^•^OH, CO_3_^•^^−^, ^1^O_2_, ^3^CDOM* and the direct photolysis (d.p.) to the photodegradation of APAP, for different values of the DOC. (**b**) DOC trends of the first order photodegradation rate constants of APAP, accounted for by CO_3_^•^^−^ and ^3^CDOM*. In both cases, it was DOC × *d*_th_ = 45 m mg_C_ L^−1^.

The reason behind the trend with a minimum of *k*_APAP_ vs. DOC is a shift in the prevailing photochemical degradation pathway of APAP, from CO_3_^•−^ at low DOC to ^3^CDOM* at high DOC (see Figure 4a). Indeed, the first-order rate constant of APAP photodegradation by CO_3_^•−^ is predicted to decrease with increasing DOC, while the ^3^CDOM* rate constant would increase with DOC (see Figure 4b). The combined degradation trends by CO_3_^•−^ and ^3^CDOM* can account well for the minimum of APAP photodegradation kinetics (compare Figure 3a and Figure 4b).

**Figure 5:**
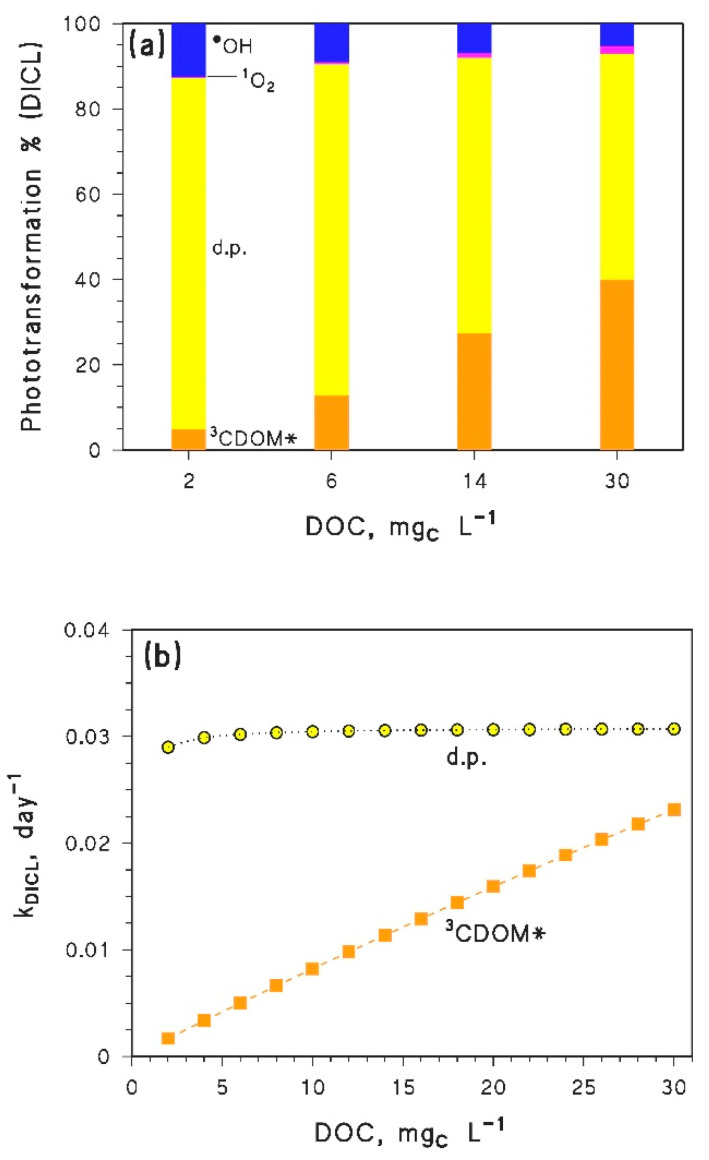
(**a**) Modeled contributions of ^•^OH, ^1^O_2_, ^3^CDOM* and the direct photolysis (d.p.) to the photodegradation of DICL, for different values of the DOC. (**b**) DOC trends of the first-order photodegradation rate constants of DICL, accounted for by direct photolysis (d.p.) and ^3^CDOM*. In both cases, it was DOC × *d*_th_ = 45 m mg_C_ L^−1^.

In the case of DICL the predicted photodegradation would be fast as well, with half-life times in the range of 8–30 days. Moreover, the first-order degradation rate constants show a continuous increase with increasing DOC (Figure 3b): this means that a long-term increase of the DOC due to browning would slightly enhance photodegradation of DICL in epilimnion water during summertime. The direct photolysis is predicted to be the main DICL photodegradation pathway (Figure 5a), which is fully consistent with literature findings [23,35]. However, the relative importance of degradation by ^3^CDOM*, which is negligible at low DOC, would become competitive at high DOC levels (Figure 5a).

Interestingly, the photodegradation rate constant of DICL by direct photolysis would practically not change with varying DOC (see Figure 5b). Actually, radiation absorption by DICL in lake water would be decreased by the fact that other lake-water components absorb sunlight as well, thereby inhibiting DICL direct photolysis [23]. However, at constant DOC × *d*_th_ the photon flux absorbed by the water column would not be modified (see Equation (12)). Therefore, the inhibition effect carried out by other water components on DICL photolysis is not expected to change much with increasing DOC, if DOC × *d*_th_ is constant. The combination of a practically constant kinetics of direct photolysis with an increasing trend of photodegradation by ^3^CDOM* (Figure 5b) accounts for the observed trend of DICL photodegradation as a function of the DOC, reported in Figure 3b.

In the case of ACEK, the predicted half-life times are considerably longer compared to the other compounds (Figure 3c). This result agrees with field data of ACEK persistence in surface waters [37,38]. Because lifetimes are so long, ACEK would undergo very limited photodegradation in the epilimnion during summer stratification. ACEK is a typical pollutant that is photodegraded by ^•^OH only [25], and in fact the DOC trend of ACEK photodegradation (Figure 3c) closely reflects the behavior of the steady-state [^•^OH] (Figure 2). Moreover, because the second-order reaction rate constant between ACEK and ^•^OH ($k_{ACEK+{}^{•}OH}$ ~ 6×10^9^ L mol^−1^ s^−1^ [25]) is about one half of the diffusion-control limit in aqueous solution [41], a pollutant that only reacts with ^•^OH could show at most twice the degradation kinetics of ACEK. Even in this upper-limit case, epilimnion photodegradation by ^•^OH during summertime would be very slow, and the relevant DOC trend would be scarcely significant.

When considering the possible impact of browning on the photodegradation kinetics of pollutants in the epilimnion of lakes, the simulation results obtained so far allow for the following general considerations to be made:

1. Compounds that are mainly degraded by direct photolysis would be poorly affected by browning, as far as photodegradation in the epilimnion is concerned. Indeed, the DOC increase would be offset by the *d*_th_ decrease, and vice versa. Compounds belonging to this class are many UV filters, some pharmaceuticals, and pesticides [42,43]. It is unfortunately very difficult to carry out a structure–activity relationship, with which to predict the importance of the direct photolysis from molecular structure information only, without experimental data [44,45].
2. Compounds that mainly/only react with ^•^OH should show a slight decrease in photodegradation kinetics because of the browning phenomenon. However, these compounds would be too photostable to be significantly degraded in the epilimnion during the summer season. More photolabile compounds, reacting through other pathways in addition to ^•^OH, could be highly affected by the DOC trend of the additional photoreactions, because [^•^OH] does not vary much with varying DOC (see Figure 2).
3. Inhibition of epilimnion photodegradation due to browning is predicted for compounds that mainly react with CO_3_^−^. However, because the reduction potential of CO_3_^•−^ is lower than the reduction potentials of many ^3^CDOM* [4], it is very difficult to find compounds that react with CO_3_^•−^ at low DOC and do not react with ^3^CDOM* (or ^1^O_2_) at high DOC. Therefore, most pollutants that react fast with CO_3_^•−^ (e.g., phenols, anilines, sulphur-containing compounds [8,46,47]) are expected to show a minimum in their epilimnion photodegradation rate constants, in a similar way as APAP. The position of the minimum would depend on the respective values of the second-order rate constants $k_{S+CO_{3}^{•-}}$ and $k_{S+{}^{3}CDOM*}$, and on the irradiance and spectrum of sunlight.
4. Because of the above considerations, in many cases the browning of medium- to high-DOC waters would accelerate the epilimnion photodegradation kinetics of pollutants, especially when photodegradation is quite fast. Indeed, despite the fact that browning makes water more colored, and thus less conducive to sunlight penetration [21], the combination of a shallower thermocline with the photoreaction pathways occurring at high (C)DOM (^3^CDOM* and, where applicable, ^1^O_2_) would speed-up photodegradation in the epilimnion.

### 3.2. Whole-Lake Photodegradation (Epilimnion + Hypolimnion)

The above considerations apply to the epilimnion where, at equal DOC × *d*_th_, increasing DOC means shallower *d*_th_. Therefore, it is assumed that the epilimnion accounts for an increasingly limited fraction of the overall lake-water volume if the DOC increases [19]. Although the overall epilimnion photochemistry may be enhanced, an increasing fraction of the lake would be in the dark (hypolimnion) as the DOC gets higher. Pollutants occurring only in the hypolimnion (e.g., because they reach the lake water through groundwater) are largely protected from photodegradation [11], and the scenario is exacerbated if the hypolimnion accounts for larger fraction of the lake.

If a compound is mostly transformed by direct photolysis and is distributed evenly in the water column before the onset of stratification, browning is expected to be detrimental to photodegradation in the whole lake volume. Indeed, the epilimnion photodegradation kinetics would not change with increasing DOC, but *d*_th_ would decrease and the hypolimnion would become deeper. According to the Lambert–Beer law, the sunlight photon flux incident on the hypolimnion ($P_{o}^{hypo}$) would not change at constant DOC × *d*_th_ (Equation (13)) [32]

(13) $$P_{o}^{hypo}=\int_{\lambda }p{}^{\circ}(\lambda )\;10^{-100A_{1}(\lambda )\;DOC\;d_{th}}d\lambda$$

However, if *d*_th_ is shallower and the hypolimnion is deeper, the same incident photon flux $P_{o}^{hypo}$ is absorbed in a higher hypolimnion volume, and photodegradation slows down (it is a similar scenario to that of a deeper lake, receiving the same sunlight irradiance as a shallower lake [27]). Moreover, the higher hypolimnion volume weights more, when the lake finally undergoes overturn and pollutant concentration is homogenized again [11,20]. In such a scenario one expects unchanged epilimnion photochemistry with increasing DOC, slower photodegradation in the hypolimnion, and a higher fraction of the lake volume accounted for by the hypolimnion. Overall, photodegradation kinetics in the whole lake volume would get slower as the DOC increases.

A less straightforward scenario would be that of a pollutant, the photodegradation of which gets faster in the epilimnion as the DOC increases. Among the studied compounds, APAP was that showing the steepest increase of the photodegradation rate constant in the epilimnion with increasing DOC, despite the low-DOC minimum (see Figure 3). Basically, the steep increase is due to the fact that APAP is the studied compound with the highest value of $k_{S+{}^{3}CDOM*}$ (Table 1).

**Figure 6:**
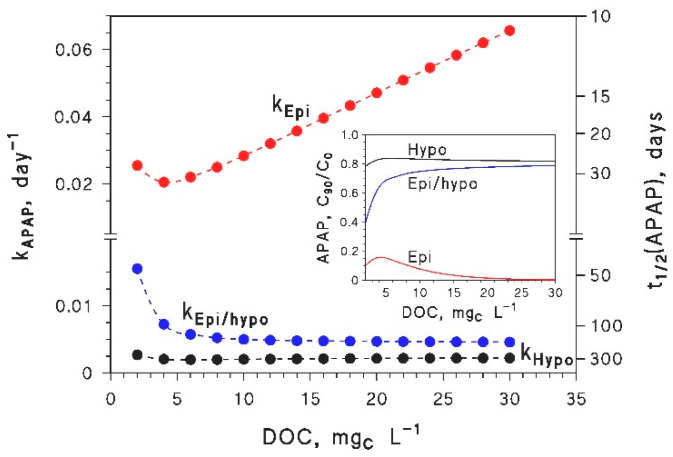
Trends of the photodegradation rate constants of APAP in the lake epilimnion (*k*_Epi_), hypolimnion (*k*_Hypo_), and whole water column (*k*_Epi/hypo_, average depth *d*_o_ = 40 m). The Epi/hypo kinetics was averaged as per Equation (**14**). In the photochemical model, to account for the sunlight intensity incident over the hypolimnion, the value of *p*°(λ) (spectral photon flux density at the water surface) was replaced by $p{}^{\circ}(\lambda )\;10^{-100A_{1}(\lambda )\;DOC\;d_{th}}\;$ that accounts for sunlight absorption in the overlying epilimnion, with DOC × *d*_th_ = 45 m mg_C_ L^−1^ and $A_{1}(\lambda )=0.45\;e^{-0.015\;\lambda }$. **Figure insert:** Concentration values of APAP after photodegradation for 90 days (C_90_), with respect to the initial APAP concentration (*C*_0_), in the epilimnion (Epi), hypolimnion (Hypo), and the whole water column (Epi/hypo). The latter was averaged as per Equation (**15**), by assuming complete lake overturn at day 90. The ratio *C*_90_/*C*_0_ is plotted as a function of the DOC, assuming DOC × *d*_th_ = 45 m mg_C_ L^−1^, and *d*_o_ = 40 m as the average depth of the lake. Other water conditions for all simulations: 10^−4^ mol L^−1^ NO_3_^−^, 10^−6^ mol L^−1^ NO_2_^−^, 10^−3^ mol L^−1^ HCO_3_^−^, 10^−5^ mol L^−1^ CO_3_^2−^. Photodegradation kinetics assumed mid-July irradiation conditions [27].

The photodegradation kinetics of APAP in epilimnion, hypolimnion and the whole lake are reported in Figure 6, as a function of the DOC, for DOC × *d*_th_ = 45 m mg_C_ L^−1^, and with *d*_o_ = 40 m as the average depth of the lake (the average depth of the hypolimnion is thus *d*_o_–*d*_th_). For the photodegradation rate constants in the epilimnion, see Figure 3a with DOC × *d*_th_ = 45 m mg_C_ L^−1^ and the related discussion. Photodegradation in the hypolimnion is very slow, which excludes effective transformation during stratification. The trend of the hypolimnion rate constant has a shallow minimum for DOC = 6 mg_C_ L^−1^, which reflects the contributions of CO_3_^•−^ and ^3^CDOM*. The average photodegradation rate constant in the whole lake (epilimnion + hypolimnion) was calculated as the average of epilimnion and hypolimnion rate constants, weighted for the respective volumes [11]

(14) $$k_{Epi/hypo}=\frac{k_{Epi}\;V_{Epi}+k_{Hypo}V_{Hypo}}{V_{Epi}+V_{Hypo}}=\frac{k_{Epi}\;d_{th}+k_{Hypo}(d_{o}-d_{th})}{d_{o}}$$

The value of *k*_Epi/hypo_ initially decreases with DOC, and then reaches a plateau. The low-DOC decrease of *k*_Epi/hypo_ (Figure 6) is due to decreasing *k*_Epi_ and to increasing volume fraction of the hypolimnion: indeed, as the DOC gets higher, *d*_th_ gets shallower, and *d*_o_–*d*_th_ increases. In contrast, increasing *k*_Epi_ after the minimum is almost completely offset by the increasing volume fraction of the hypolimnion, and *k*_Epi/hypo_ reaches a plateau. When *k*_Epi_ increases with DOC, as for APAP at high DOC, browning would not modify the average whole-lake photodegradation kinetics by much.

Still, the occurrence of a shallow epilimnion characterized by fast photoreactions plus a large, poorly reactive hypolimnion is not favorable to photodegradation. The issue is that the pollutant may be totally degraded in the epilimnion, after which its degradation has to stop for lack of substrate, even if the reactive transient species responsible for photodegradation are still photogenerated. At the same time, poor photodegradation takes place in the hypolimnion [11].

To account for this, APAP was assumed to be initially distributed evenly in the water column (uniform initial concentration, *C*_0_). No further immission of APAP in the lake water was assumed to take place thereafter, so that APAP concentration would only change due to degradation. Stratification was assumed to last for 90 days, after which complete lake overturn would occur. Before overturn, independent photodegradation would take place in the epilimnion and hypolimnion, and the time evolution of the respective concentrations would follow the trends (*C*_t_)_Epi_ = *C*_0_ exp(^−^*k*_Epi_ × *t*), and (*C*_t_)_Hypo_ = *C*_0_ exp(^−^*k*_Hypo_ × *t*). The concentration values *C*_90_ for *t* = 90 days are reported in the insert of Figure 6, as *C*_90_/*C*_0_ as a function of the DOC, with DOC × *d*_th_ = 45 m mg_C_ L^−1^ and *d*_o_ = 40 m. The average value of *C*_90_ in the whole lake was calculated as the weighted average of the epilimnion and hypolimnion concentrations, as [11]

(15) $${\left(C_{90}\right)}_{Epi/hypo}=\frac{{\left(C_{90}\right)}_{Epi}\;V_{Epi}+{\left(C_{90}\right)}_{Hypo}V_{Hypo}}{V_{Epi}+V_{Hypo}}=\frac{{\left(C_{90}\right)}_{Epi}\;d_{th}+{\left(C_{90}\right)}_{Hypo}(d_{o}-d_{th})}{d_{o}}$$

The results (Figure 6, insert) show extensive APAP photodegradation in 90 days in the lake epilimnion, with (*C*_90_)_Epi_ ~ 0 for DOC > 15–20 mg_C_ L^−1^. In contrast, limited degradation (~20%) would occur in the hypolimnion, and the resulting whole-lake concentration ((*C*_90_)_Epi/hypo_) would increase with increasing DOC. Therefore, the occurrence of a shallow epilimnion at high DOC would be detrimental to APAP photodegradation, despite elevated epilimnion reactivity.

## 4. Conclusions

Lake-water browning, for which climate change is an important driver, is expected to affect the summer stratification of lakes by decreasing the depth of the thermocline. The opposite phenomenon would take place in some environments, where the effects of climate change would run in the contrary direction. In the case of browning, the occurrence of a shallower and CDOM-richer epiliminon has the potential to alter the photodegradation kinetics of pollutants. The effect may be null, positive, or negative, depending on the distribution of the pollutant within the water column and on its photodegradation pathway(s).

If a pollutant is confined into the lake epilimnion during summer stratification, e.g., because it reaches the lake surface by runoff, the effect of browning depends on the main photodegradation pathway(s). Compounds that are mainly degraded by direct photolysis would be hardly affected by browning, because the decreasing depth of the thermocline offsets the CDOM increase. Browning would slightly slow down photodegradation of compounds that react predominantly or exclusively with ^•^OH. However, their photodegradation would be too slow to be really significant during summer stratification. Increasing (C)DOM would be highly detrimental to CO_3_^•−^ photodegradation, but would favor the processes triggered by ^3^CDOM* and ^1^O_2_. Because it is hard to find a compound that reacts fast with CO_3_^•−^ (typical examples are phenols and anilines) and is poorly reactive with ^3^CDOM*, in these cases one expects a minimum in the photodegradation rate constant as a function of the DOC. The minimum would be due to the combination of decreasing degradation by CO_3_^•−^ and increasing degradation by ^3^CDOM* (and possibly ^1^O_2_). If the initial DOC is high enough, browning can considerably enhance photodegradation kinetics by ^3^CDOM* in the epilimnion water.

If the whole water column (epilimnion + hypolimnion) is considered, in which a pollutant is initially evenly distributed, the occurrence of a shallow epilimnion is always detrimental to photodegradation. It affects either the kinetics of photoreactions or, even at unchanged kinetics, the way the pollutant is photodegraded, making degradation less effective. In this case, if a pollutant is initially distributed within the whole lake volume, browning will always inhibit photodegradation during the summer stratification period.

## Figures and Tables

**Table 1:** Photoreactivity parameters of paracetamol (S = APAP), diclofenac (S = DICL), and acesulfame K (S = ACEK). Note that Φ is the symbol for the direct photolysis quantum yield.

Parameter	Paracetamol (APAP)	Diclofenac (DICL)	Acesulfame K (ACEK)
Φ_S_, unitless	4.6 × 10^−2^	9.4 × 10^−2^	5.5 × 10^−3^
$k_{S+{}^{•}OH}$, L mol^−1^ s^−1^	1.9 × 10^9^	9.3 × 10^9^	5.9 × 10^9^
$k_{S+{{CO}_{3}^{•}}^{-}}$, L mol^−1^ s^−1^	3.8 × 10^8^	Negligible	Negligible
$k_{S+{}^{1}O_{2}}$, L mol^−1^ s^−1^	3.7 × 10^7^	1.3 × 10^7^	2.8 × 10^4^
$k_{S+{}^{3}CDOM*}$, L mol^−1^ s^−1^	1.6 × 10^9^	6.4 × 10^8^	Negligible

**Table 2:** Comparison between field data and model predictions for the phototransformation kinetics of paracetamol (APAP), diclofenac (DICL), and acesulfame K (ACEK).

Parameter	Field Lifetime, Days	Location	Modeled Lifetime, Days
APAP	1.5–2.5 [36]	Tokushima (Japan)	2.5
DICL	8.3 [35]	Greinfensee (Switzerland)	7–8
ACEK	>1200 [37]	Norra Bergundasjön (Sweden)	>700
